# Imported Schistosomiasis, China, 2010–2018

**DOI:** 10.3201/eid2601.191250

**Published:** 2020-01

**Authors:** Si-Min Dai, Zhou Guan, Li-Juan Zhang, Shan Lv, Chun-Li Cao, Shi-Zhu Li, Jing Xu

**Affiliations:** National Institute of Parasitic Diseases, Chinese Center for Disease Control and Prevention, Shanghai, China; World Health Organization Collaborating Centre for Tropical Diseases, Shanghai; National Tropical Disease Research Center, Shanghai

**Keywords:** schistosomiasis, imported case, China, parasites, snails, Biomphalaria straminea

## Abstract

China has made remarkable progress in reducing schistosomiasis caused by *Schistosoma japonicum* over the past 7 decades but now faces a severe threat from imported schistosomiasis. Results from national surveillance during 2010–2018 indicate integrating active surveillance into current surveillance models for imported cases is urgently needed to achieve schistosomiasis elimination in China.

Six species of *Schistosoma* trematodes are known to infect humans, but only *S. japonicum* is prevalent in China (*1**,**2*). However, because of enhanced international cooperation and communication, 2 *Schistosoma* species were identified from schistosomiasis cases imported to China: *S. haematobium* and *S. mansoni*. In addition, the *Biomphalaria straminea* snail, an intermediate host of *S. mansoni* trematodes, has been introduced into Shenzhen, Guangdong Province (*3*,*4*), so the possibility exists that imported *S. mansoni* trematodes could be transmitted to these snails and result in locally acquired human infections. Therefore, we analyzed imported schistosomiasis status in China through data collected from the National Notifiable Disease Report System (NNDRS) during 2010–2018 to provide information for national policy making on schistosomiasis elimination.

We defined imported schistosomiasis as schistosomiasis in an endemic focus outside mainland China. A confirmed imported case had to meet both of the following diagnostic criteria: the patient was given a diagnosis of schistosomiasis, meaning that schistosome eggs or miracidia were detected in the patient’s biological samples; the patient once traveled to and/or worked in schistosomiasis-endemic areas in other countries during transmission seasons (Table).

**Table:** Imported schistosomiasis, mainland China, 2010–2018*

Diagnosis year	Reported province	Patient age, y	Source country	Activity/occupation	Parasite
2018	Jiangsu	45	Sudan	Businessman	*Schistosome haematobium*
2018	Jiangsu	60	Sudan	Worker	*S. haematobium*
2018	Jiangsu	55	Angola	Worker	*S. haematobium*
2018	Jiangsu	49	Angola	Worker	*S. haematobium*
2016	Beijing	34	Uganda	Civil servant	*S. mansoni*
2016	Zhejiang	58	Nigeria	Farmer	*S. haematobium*
2016	Zhejiang	56	Nigeria	Farmer	*S. haematobium*
2016	Fujian	35	Angola	Housework	*S. haematobium*
2016	Jiangxi	51	Zambia	Worker	*S. haematobium*
2016	Sichuan	40	Unknown	Businessman	*S. haematobium*
2015	Beijing	68	Cameroon	Retiree	Unknown
2015	Beijing	35	Nigeria	Businessman	*S. mansoni*
2015	Zhejiang	57	Nigeria	Worker	*S. haematobium*
2015	Guangxi	45	Angola	Seafarer	*S. haematobium*
2014	Shandong	43	Angola	Farmer	*S. haematobium*
2012	Fujian	30	Unknown	Businessman	*S. haematobium*
2012	Shanxi	46	Mali	Worker	Unknown
2012	Shanxi	60	Angola	Worker	Unknown
2010	Shanxi	56	Zambia	Worker	Unknown
2010	Shanxi	55	Zambia	Worker	Unknown
2010	Shanxi	39	Zambia	Worker	Unknown
2010	Shanxi	42	Congo	Worker	Unknown

*All cases occurred in men.

Twenty-two cases were reported during the study period. All imported cases occurred in men; their ages ranged from 30 to 68 years (mean 48 ± SD 9.98 years). Nineteen (86%) were 30–58 years of age, and 3 (14%) were >58 years of age. Half of them were workers who returned to China from Africa; 4 (18%) were businessmen. No case was reported in Guangdong Province; 4 cases were reported in the neighboring provinces of Guangxi, Jiangxi, and Fujian.

The 22 cases acquired *Schistosoma* infection in 8 countries in Africa. Of the 20 case-patients for whom detailed data were available, most were infected in central Africa (Angola, 6 [30%] cases) and Cameroon and Congo (1 [5%] case each). In West Africa, Nigeria accounted for 6 (30%) cases and Mali for 1 (5%) case; in southern Africa, Zambia accounted for 4 (20%) cases.

More than 1 million persons from China are estimated to work in Africa (*5*). However, during the study period, only 22 confirmed cases were recognized. Considering the high risk for infection in Africa and the existing passive surveillance system, the number of imported schistosomiasis cases might be substantially underestimated.

As a national schistosomiasis surveillance system in China, NNDRS mirrors the local and imported case status passively reported on a national scale: when patients exposed in Africa had symptoms after returning to China, they sought treatment at hospitals on their own. If schistosomiasis was confirmed, the physician reported it to NNDRS, the only case report source in the existing surveillance system. However, this surveillance model has limitations. For example, cases might be misdiagnosed and missed because medical staff have insufficient clinical awareness of African schistosomiasis or because some patients may be asymptomatic or have mild symptoms.

Another challenge is that global integration has contributed to an increasing number of persons from China who travel abroad, and most persons who travel to Africa are not equipped with essential knowledge about schistosomiasis prevention and control. In addition, introduction of the *B. straminea* snail into southern China increases the risk that persons in China might acquire *S. mansoni* infection.

Because imported schistosomiasis may increase the disease burden in China and hinder the process of schistosomiasis elimination, several measures are urgently needed. First, a well-rounded active surveillance system needs to be integrated into the current monitoring model to ensure an accurate and quick response. When performing active surveillance through screening, sensitive and rapid immunologic tests should be widely conducted among the most susceptible group before etiologic examinations are conducted. Our results suggest that male workers returning from Africa are the most susceptible population. Meanwhile, because the intermediate host of African schistosomiasis, the *B. straminea* snail, has been introduced to Guangdong Province in southern China, persons returning from *S. mansoni*–endemic countries or regions. Guangdong Province and adjacent provinces should be a primary target population for surveillance. Identifying migrant workers as the highest priority group for active surveillance will be of great value. Second, China should collaborate with the World Health Organization and other relevant international institutions to evaluate the feasibility of existing serologic detection kits developed by China for diagnosis of imported schistosomiasis or develop new kits if necessary. Third, training programs on standardized clinical diagnostic criteria and normative case reporting operation are required in different types of healthcare organizations (e.g., hospitals, Centers for Disease Control and Prevention, entry and exit border stations) in mainland China to ensure accurate diagnosis and disease treatment. Other measures, such as multisector cooperation and coordination, could help achieve elimination of schistosomiasis in China.
